# Supplementary material for: Sense of Agency and Skills Learning in Virtual-Mediated Environment: A Systematic Review
Source: Brain Sci. 2024 Mar 31;14(4):350. doi: 10.3390/brainsci14040350 (PMC11048251; doi:10.3390/brainsci14040350)
Supplement: Supplementary file 1 [file brainsci-14-00350-s001.zip › brainsci-2905255-supplementary.pdf]

Supplementary Material

Supplementary Table S1: Preferred Reporting Items for Systematic Reviews and Meta-Analyses (PRISMA) guidelines

Section and Topic	Item #	Checklist item	Location where item is reported
**TITLE**			
Title	1	Identify the report as a systematic review.	p 1
**ABSTRACT**			
Abstract	2	See the PRISMA 2020 for Abstracts checklist.	p 1
**INTRODUCTION**			
Rationale	3	Describe the rationale for the review in the context of existing knowledge.	pp 1-3
Objectives	4	Provide an explicit statement of the objective(s) or question(s) the review addresses.	p 3
**METHODS**			
Eligibility criteria	5	Specify the inclusion and exclusion criteria for the review and how studies were grouped for the syntheses.	pp 3,4; Table 1
Information sources	6	Specify all databases, registers, websites, organisations, reference lists and other sources searched or consulted to identify studies. Specify the date when each source was last searched or consulted.	pp 3,4; Supplementary Table S2
Search strategy	7	Present the full search strategies for all databases, registers and websites, including any filters and limits used.	pp 3,4; Supplementary Table S2

Section and Topic	Item #	Checklist item	Location where item is reported
Selection process	8	Specify the methods used to decide whether a study met the inclusion criteria of the review, including how many reviewers screened each record and each report retrieved, whether they worked independently, and if applicable, details of automation tools used in the process.	pp 3,4
Data collection process	9	Specify the methods used to collect data from reports, including how many reviewers collected data from each report, whether they worked independently, any processes for obtaining or confirming data from study investigators, and if applicable, details of automation tools used in the process.	pp 3,4
Data items	10a	List and define all outcomes for which data were sought. Specify whether all results that were compatible with each outcome domain in each study were sought (e.g. for all measures, time points, analyses), and if not, the methods used to decide which results to collect.	pp 3,4; Supplementary Table S2
	10b	List and define all other variables for which data were sought (e.g. participant and intervention characteristics, funding sources). Describe any assumptions made about any missing or unclear information.	pp 3,4, Table 1 Supplementary Table S3; Supplementary Table S4.
Study risk of bias assessment	11	Specify the methods used to assess risk of bias in the included studies, including details of the tool(s) used, how many reviewers assessed each study and whether they worked independently, and if applicable, details of automation tools used in the process.	pp 4,5; Supplementary Table S3; Supplementary Table S4.
Effect measures	12	Specify for each outcome the effect measure(s) (e.g. risk ratio, mean difference) used in the synthesis or presentation of results.	Table 2; Table 3; Supplementary Table S5
Synthesis methods	13a	Describe the processes used to decide which studies were eligible for each synthesis (e.g. tabulating the study intervention characteristics and comparing against the planned groups for each synthesis (item #5)).	Table 1
	13b	Describe any methods required to prepare the data for presentation or synthesis, such as handling of missing summary statistics, or data conversions.	NA
	13c	Describe any methods used to tabulate or visually display results of individual studies and syntheses.	pp 5-6

Section and Topic	Item #	Checklist item	Location where item is reported
	13d	Describe any methods used to synthesize results and provide a rationale for the choice(s). If meta-analysis was performed, describe the model(s), method(s) to identify the presence and extent of statistical heterogeneity, and software package(s) used.	pp 5-6; Table 2; Table 3; Supplementary Table S5
	13e	Describe any methods used to explore possible causes of heterogeneity among study results (e.g. subgroup analysis, meta-regression).	NA
	13f	Describe any sensitivity analyses conducted to assess robustness of the synthesized results.	NA
Reporting bias assessment	14	Describe any methods used to assess risk of bias due to missing results in a synthesis (arising from reporting biases).	Supplementary Table S3; Supplementary Table S4
Certainty assessment	15	Describe any methods used to assess certainty (or confidence) in the body of evidence for an outcome.	NA
**RESULTS**			
Study selection	16a	Describe the results of the search and selection process, from the number of records identified in the search to the number of studies included in the review, ideally using a flow diagram.	pp 5-6; Figure 1
	16b	Cite studies that might appear to meet the inclusion criteria, but which were excluded, and explain why they were excluded.	pp 5-6; Figure 1
Study characteristics	17	Cite each included study and present its characteristics.	pp 5-13; Figure 2; Table 2; Table 3; Supplementary Table S5
Risk of bias in studies	18	Present assessments of risk of bias for each included study.	p 6; Supplementary

Section and Topic	Item #	Checklist item	Location where item is reported
			Table S3; Supplementary Table S4;
Results of individual studies	19	For all outcomes, present, for each study: (a) summary statistics for each group (where appropriate) and (b) an effect estimate and its precision (e.g. confidence/credible interval), ideally using structured tables or plots.	Supplementary Table S5
Results of syntheses	20a	For each synthesis, briefly summarise the characteristics and risk of bias among contributing studies.	pp 5-13; Table 2, Table 3; Supplementary Table S3; Supplementary Table S4
	20b	Present results of all statistical syntheses conducted. If meta-analysis was done, present for each the summary estimate and its precision (e.g., confidence/credible interval) and measures of statistical heterogeneity. If comparing groups, describe the direction of the effect.	pp 5-13; Supplementary Table S 5
	20c	Present results of all investigations of possible causes of heterogeneity among study results.	NA
	20d	Present results of all sensitivity analyses conducted to assess the robustness of the synthesized results.	NA
Reporting biases	21	Present assessments of risk of bias due to missing results (arising from reporting biases) for each synthesis assessed.	p 6; Supplementary Table S3; Supplementary Table S4
Certainty of evidence	22	Present assessments of certainty (or confidence) in the body of evidence for each outcome assessed.	NA
**DISCUSSION**			

Section and Topic	Item #	Checklist item	Location where item is reported
Discussion	23a	Provide a general interpretation of the results in the context of other evidence.	pp 13-16
	23b	Discuss any limitations of the evidence included in the review.	P 16
	23c	Discuss any limitations of the review processes used.	P 16
	23d	Discuss implications of the results for practice, policy, and future research.	pp 16-17
**OTHER INFORMATION**			
Registration and protocol	24a	Provide registration information for the review, including register name and registration number, or state that the review was not registered.	p 3
	24b	Indicate where the review protocol can be accessed, or state that a protocol was not prepared.	p 3
	24c	Describe and explain any amendments to information provided at registration or in the protocol.	p 3
Support	25	Describe sources of financial or non-financial support for the review, and the role of the funders or sponsors in the review.	p 17
Competing interests	26	Declare any competing interests of review authors.	p 17
Availability of data, code and other materials	27	Report which of the following are publicly available and where they can be found: template data collection forms; data extracted from included studies; data used for all analyses; analytic code; any other materials used in the review.	NA

Supplementary Table S2: Search steps for MEDLINE and SCOPUS

Database		Query	Research in	Items found up to June 22
**PubMed**	#1	"implicit learning" OR "perceptual learning" OR "skill acquisition" OR "skill learning" OR "procedural learning" OR "implicit knowledge" OR skills OR "motor learning" OR "motor control" OR "sequence learning" OR "motor sequence learning"	Title/Abstract	208,389
	#2	“agency" OR "sense of agency" OR "motor embodiment" OR "embodiment"	Title/Abstract	166,414
	#3	Combine #1 AND #2		2,073
	#4	Limit to "Human" "English"		991
**Scopus**	#1	(TITLE-ABS-KEY "implicit learning" OR "perceptual learning" OR "skill acquisition" OR "skill learning" OR "procedural learning" OR "implicit knowledge" OR “skills” OR "motor learning" OR "motor control" OR "sequence learning" OR "motor sequence learning") AND (TITLE-ABS-KEY agency OR "sense of agency" OR "embodiment")	Title/Abstract/Keywords	11,236
	#2	Limit to "Human" and "English"<br>Exclude "bioc" AND "agri" "envi" AND "nurs" OR "phar" OR "busi" AND "immu" OR “dent” OR “vete” OR “phys”		1,682

Supplementary Table S3: Risk of bias in within subject studies

	Appropriate cross-over design	Randomised treatment order	Carry-over effect	Unbiased data	Allocation concealment	Blinding	Incomplete outcome data	Selective outcome reporting	Other bias
Adamovich et al., 2009 [42]	-	?	?	-	?	+	-	-	-
Aoyagi et al., 2021 [43]	-	-	-	-	?	+	-	-	-
Haar et al., 2021 [44]	-	?	-	-	?	-	-	-	+
Kannape et al., 2010 [45]	-	-	?	-	?	+	-	-	-
Kumar and Srinivasan, 2017 [46]	-	-	?	-	?	+	-	-	-
Metcalfe et al., 2013 [47]	-	-	?	-	?	+	-	-	-
Nataraj et al., 2020a [48]	-	?	-	-	?	+	-	-	+
Nataraj et al., 2020b [49]	-	?	-	-	?-	+	-	-	-
Ozen et al., 2019 [50]	-	-	-	-	-	+	-	-	+

Padrao et al., 2015 [51]	-	?	?	-	?	+	-	-	-
Ratcliffe and Newport, 2017 [52]	-	-	-	-	?	+	-	-	-
Rognini et al., 2013 [53]	-	-	?	-	?	+	-	-	-
Tidoni et al., 2014 [54]	-	+	?	-	?	+	-	-	+
Weibel et al., 2015 [55]	-	-	?	-	?	+	-	-	-

-: low; ?: unclear; +: high risk of bias

Supplementary Table S4: CASP Randomised Controlled Trial Standard Checklist

**CASP Randomised Controlled Trial Standard Checklist**	Ozen et al., 2021 [56]
**1. Did the study address a clearly focused research question?**<br>*CONSIDER:*<br>*Was the study designed to assess the outcomes of an intervention?*<br>*Is the research question 'focused' in terms of:*<br>• *Population studied*<br>• *Intervention given*<br>• *Comparator chosen*<br>• *Outcomes measured?*	yes
**2. Was the assignment of participants to interventions randomised?** *CONSIDER:*<br>● *How was randomisation carried out? Was the method appropriate?*<br>● *Was randomisation sufficient to eliminate systematic bias?*<br>● *Was the allocation sequence concealed from investigators and participants?*	yes
**3. Were all participants who entered the study accounted for at its conclusion?**<br>*CONSIDER:*	no

● *Were losses to follow-up and exclusions after randomisation accounted for?*<br>● *Were participants analysed in the study groups to which they were randomised (intention-to-treat analysis)?*<br>● *Was the study stopped early? If so, what was the reason?*	
**4.**<br>**• Were the participants 'blind' to intervention they were given?**<br>**• Were the investigators 'blind' to the intervention they were giving to participants?**<br>**• Were the people assessing/analysing outcome/s 'blinded'?**	no
**5. Were the study groups similar at the start of the randomised controlled trial?**<br>*CONSIDER:*<br>● *Were the baseline characteristics of each study group (e.g. age, sex, socio-economic group) clearly set out?*<br>● *Were there any differences between the study groups that could affect the outcome/s?*	yes
**6. Apart from the experimental intervention, did each study group receive the same level of care (that is, were they treated equally)?** *CONSIDER:*<br>*• Was there a clearly defined study protocol? • If any additional interventions were given (e.g. tests or treatments), were they similar between the study groups?*<br>*• Were the follow-up intervals the same for each study group?*	yes

**7. Were the effects of intervention reported comprehensively?**<br>*CONSIDER:*<br>• *Was a power calculation undertaken?*<br>• *What outcomes were measured, and were they clearly specified?*<br>• *How were the results expressed? For binary outcomes, were relative and absolute effects reported?*<br>• *Were the results reported for each outcome in each study group at each follow-up interval?*<br>• *Was there any missing or incomplete data?*<br>• *Was there differential drop-out between the study groups that could affect the results?*<br>• *Were potential sources of bias identified?*<br>• *Which statistical tests were used?*<br>• *Were p values reported?*	yes
**8. Was the precision of the estimate of the intervention or treatment effect reported?**<br>*CONSIDER:*<br>• *Were confidence intervals (CIs) reported?*	no
**9. Do the benefits of the experimental intervention outweigh the harms and costs?**<br>*CONSIDER:*<br>• *What was the size of the intervention or treatment effect?*<br>• *Were harms or unintended effects reported for each study group?*<br>• *Was a cost-effectiveness analysis undertaken? (Cost-effectiveness analysis allows a comparison to be made between different interventions used in the care of the same condition or problem.)*	Can’t tell

**10. Can the results be applied to your local population/in your context?**<br>*CONSIDER:*<br>• *Are the study participants similar to the people in your care?*<br>• *Would any differences between your population and the study participants alter the outcomes reported in the study?*<br>• *Are the outcomes important to your population?*<br>• *Are there any outcomes you would have wanted information on that have not been studied or reported?*<br>• *Are there any limitations of the study that would affect your decision?*	yes
**11. Would the experimental intervention provide greater value to the people in your care than any of the existing interventions?**<br>*CONSIDER:*<br>• *What resources are needed to introduce this intervention taking into account time, finances, and skills development or training needs?*<br>• *Are you able to disinvest resources in one or more existing interventions in order to be able to re-invest in the new intervention?*	yes

Supplementary Table S5: Synthesis of main outcomes

	Objectives	Number of participants	Design	Paradigm	Experimental Procedures	Behavioral, neuronal, and psychometric outcomes	Main findings
Adamovich et al., 2009 [25]	To develop a VR compatible with fMRI allowing simultaneous assessment of motor behavior and brain activity and to delineate how these work and interact in VR.	13 subjects	within-subjects design	Experimental Feedback	Subjects wear a 5DT data glove on their hand to control the movement of a virtual hand model (finger movement task).<br><br>4 conditions:<br>• OTI (to observe the virtual hand flex the index-middle-ring-pinky fingers in randomly defined sequential order. The virtual hand looks like a real hand);<br>• MOVE_h (to execute the observed sequence, receiving real-time visual feedback of the subject's real movement through the virtual hand);<br>• WATCH_e (to observe ellipsoid, that matched the virtual hand in size, color, and movement frequency);<br>• MOVE_e (to execute a previously observed finger sequence).	• fMRI recordings;<br>• Finger kinematics (joint angle excursion);<br>• Subjects control toward actions (agency).	• During observation, there is a time-dependent increase in the BOLD signal in the left posterior insula (OTI) and the right inferior occipital lobe (MOVE_h). These changes in BOLD signals occur despite there being no differences in movement kinematics observed across the blocks for both observation and execution conditions (all $p<0.001$);<br>• Activation patterns observed during the observation of movements in VR: the somatosensory and intraparietal sulcus, motor cortex, bilateral anterior insula, bilateral frontal lobes, bilateral occipital lobe, and the right anterior/posterior intermediate cerebellum (OTI); the fusiform gyrus of the temporal cortex, superior parietal lobe, anterior insula, middle frontal gyrus, and the medial frontal lobe (WATCH_e) (all $p<0.001$);<br>• During the execution of movements activations are observed in the right cerebellar cortex, postcentral gyrus and precentral gyrus, right inferior parietal lobule, and bilateral insular cortex; the angular

							gyrus, precuneus, and extrastriate body area (associated with the sense of agency);<br>● Feedback administered on VR hands correlates with activation of the right fusiform gyrus. Contrasting feedback from VR hands with ellipsoids revealed activation in bilateral angular gyri, precuneus, inferior occipital lobe, and the occipitotemporal junction (all p<0.001);<br>● Movement consistency for each finger is stable across epochs and conditions (p>0.05).
Aoyagi et al., 2021 [26]	To investigate the impact of altered visual feedback on the sense of agency over participant's movements, inducing disturbed motor control.	Exp1= 15 subjects;<br>Exp2= 14 subjects.	within-subjects design	Mismatch	EXP1<br>Participants have to move their right hand along a specified trajectory while aiming to keep a visual target as close to the trajectory as possible. The study comprises four conditions:<br>● Delayed condition: visual feedback of participants' hand positions is delayed by 600 ms;<br>● No-delay condition: the position of the visual stimulus accurately represents the real-time position of the participant's hand;<br>● Aligned condition: The visual stimulus is presented precisely at the real hand position;	EXP1<br>● Questionnaire on the sense of control of the movement;<br>● Agency rating;<br>● Motion error.<br>EXP2<br>● Agency rating;<br>● Motion error.	EXP1<br>● Main effect of delay and visual modification (p<0.001) on agency rating (both p<0.001), on motion error (p<0.001 and p p=0.013, respectively), and visual prediction error (both p<0.001)<br>EXP2<br>● Weight significantly influences the sense of agency (p=0.004);<br>● The distortion of the visual stimulus significantly affects outcomes when comparing the "weighted & aligned" and "weighted & offset" conditions (p=0.018);

					• Offset condition: the visual stimulus is presented at the midpoint between the real hand position and the nearest point on the trajectory to the hand position.<br><br>EXP2<br><br>The task is the same as the EXP1, except for the absence of delay (but a weight is attached to the right wrist of the participants to interfere with the motor control); the presence of a vertical-eight-shaped trajectory; and the presence of a target stimulus (a moving ball).<br><br>The study includes three experimental conditions:<br><br>• Baseline condition: participants experience no weight and no modifications to the visual feedback. The position of the visual stimulus is the same as the subject’s real hand;<br><br>• Weighted & aligned condition: participants experience added weight without any distortion to the visual feedback, and the visual stimulus represents the actual position of the participants' hands;<br><br>• Weighted & offset condition: participants experience added weight, and the visual stimulus is presented at the median point between the actual hand position and the target stimulus<br><br>.		• Visual prediction errors are significantly reduced in the "weighted & offset" condition compared to the "weighted & aligned" condition (p=0.001).

Haar et al., 2021 [27]	To perform a validation of an Embodied Virtual Reality (EVR) system for practical motor learning in real-world scenarios and to regulate the visual feedback for the real-world task.	10 subjects	within-subjects design	Experimental Feedback	The position of virtual billiards tables, balls, and cue sticks in VR and the world are matched and presented simultaneously. Subjects only see VE and receive tactile feedback.<br><br>The motion of the real balls belonging to the task in real words is tracked using a computer vision system. The subject has to shoot a target ball.<br><br>Participants' full-body movements are real-time tracked during motor learning using motion-tracking wireless units (IMUs).	● Trial error: absolute angular difference between the direction of the movement vector of the target ball and the expected direction to guide the target ball to the center of the pocket;<br><br>● Velocity Profile Error (VPE): minimum over the correlation between the velocity profile of each joint in a particular trial and the velocity profiles of the same joint across all successful trials;<br><br>● Intertrial variability: residuals obtained from fitting a regression line to the direction of the ball in each block.	● Improvement of success rates (p=0.007) but slower learning in VR than in the real-world task;<br><br>● Gradual decrease in the mean directional absolute error, occurring at a slower rate and to a lesser extent in the Embodied Virtual Reality environment compared to the real-world task;<br><br>● In the real-world task, the intertrial variability decreases, whereas in EVR there is not a clear observed trend;<br><br>● In the EVR there is no decrease of VPE.
Kannape et al., 2010 [28]	To find how the agency consciously monitors actions of the full body in space during locomotion.	Exp1= 9 subjects;<br><br>Exp2= 14 subjects.	within-subjects design	Mismatch	EXP1<br><br>Participants wear a suit to enable real-time animation of a virtual body shown from the back in a virtual environment using infrared red markers. They navigate the virtual body to a target (represented as a cylinder) by walking. The virtual body's walking trajectory is distorted in a randomized order, resulting in a final location that is expressed as an angle relative to an imaginary straight line towards the target and the deviation expressed by the participant to compensate for the distortion is the Motor Performance (MP).<br><br>EXP2	● Motor Awareness (MA): to indicate whether the movement of the virtual body corresponded to their real movement;<br><br>● Motor Awareness Index (MAI): the likelihood of errors in self-attribution for the gain (MP/angular deviation) in condition with an angular deviation;<br><br>● MP: the total angle compensated by the participant considering the endpoint of each of their movement trajectories.	EXP1<br><br>● According to MAI analysis, the highest values occur at angular deviations of ±5° and ±10°. More frequent errors in MA are not attributed to a decrease in MP gain at angular deviations exceeding 10°◦;<br><br>● Negative correlation between the endpoints of participants' walking paths and the degree of angular deviation introduced to the virtual body's walking trajectory ($p < 0.001$).

					The virtual body is shown in 4 different conditions (manipulating orientation and direction): “upright/back”; “upright/front”; “inverted/back”; “inverted/front”. Four main blocks are randomized for each participant (one condition per block).		EXP2<br><br>• MP is highest in the “upright/back” condition when compared to MP in the “upright/front” ($p = 0.01$) and “inverted/back” conditions ($p < 0.01$);<br><br>• No statistical differences in MA across conditions (all $p>0.18$);<br><br>• MAI: participants are significantly more<br><br>prone to attribute errors to themselves in the inverted conditions compared to the upright ones, as indicated by the main effect of Orientation ($p=0.016$).
Kumar and Srinivasan., 2017 [29]	To detect changes in motor control at multiple levels influences agency.	Exp1= 18 subjects	within-subjects design	Mismatch	EXP1<br><br>To hit a target using a joystick and then verbally estimate how much time elapses between pressing the trigger and the circle appearing on the screen; to rate the interval on a scale of 0–900 ms (100, 400, 700). The manipulation involves introducing noise into the joystick controller at different levels: low control; medium control; and full control.	• SOA: Intentional Binding;<br><br>• Hit accuracy.	EXP1<br><br>• The estimated interval is longer in the low control condition than in the medium control condition ($p = 0.03$) when the target is not hit;<br><br>• No differences between the low control and medium control conditions ($p = 1$) when the target is correctly hit ;
Kumar and Srinivasan, 2017 [29]	To detect changes in motor control at multiple levels influences agency.	Exp2a= 20 subjects;	within-subjects design	Experimental Feedback	EXP2<br>To determine the time interval between pressing the trigger and the circle appearing on the screen:	• SOA: Intentional Binding;<br><br>• Hit accuracy.	EXP2a<br><br>• Significant main effect of SOA ($p<0.001$), indicating a lower estimated interval for the 400

		Exp2b= 16 subjects.			• 2a: The circle appears in blue, and feedback is provided simultaneously with the sensory outcome of the action.<br><br>• 2b: The circle appears in either green or yellow, and feedback is given alongside the appearance of a circular disc that represents the outcome.		ms condition compared to the 700 ms condition;<br><br>• Significant main effect of target completion (p=0.014), revealing larger estimations when the target is completed compared to when it is not completed.<br><br>EXP2b;<br><br>• Main effect of SOA (p=0.002), with a lower estimated interval for 400 ms compared to 700 ms SOA;<br><br>• The main effect of the target and the interaction between target completion and SOA are not significant.
Metcalfe et al., 2013 [30]	How variations in distal outcomes and proximal actions influence people's Judgments of Agency (JOA) and Judgment of Performance (JOP).	Exp1= 21 subjects;<br><br>Exp2= 21 subjects.	within-subjects design	Mismatch	EXP1<br><br>Participants have to play a computer game using a mouse to move a box in both left or right directions by touching all the "X" (some of these targets produce a sound when touched) and avoiding all the "O". After the task, participants are required to judge their performance. In this task, the mouse is subjected to the following conditions: "turbulence" (feeling a delay between the cursor position and the mouse position); no turbulence" (the delay is absent).<br><br>EXP2	EXP1<br><br>• Proportion hit;<br><br>• JOA and JOP, by moving an indicator bar to the desired point along a marked visual analog scale.<br><br>EXP2<br><br>• Proportion exploded;<br><br>• JOA and JOP, involving the movement of an indicator bar to the desired	EXP1<br><br>• The main effect of Turbulence is significant ($p<0.001$)indicating that the hit rate is higher in the absence of turbulence compared to when turbulence is present.;<br><br>• JOAs are higher in the No Turbulence condition than the Turbulence condition ($p<0.0001$).<br><br>EXP2<br><br>• The exploding rate is higher in the Turbulence condition compared to the No

					Same computer program as in EXP1 but participants have to explode the target “X”.	point along a marked visual analog scale.	Turbulence condition ($p<0.0001$);<br>● JOAs are significantly lower in the Turbulence condition than in the No Turbulence condition ($p<0.0001$);<br>● The main effect of Turbulence on JOPs is significant ($p<0.001$).
Nataraj et al., 2020a [31]	To investigate the generalized effects of positive feedback (PF) vs negative<br><br>feedback (NF) on performative indices and the sense of agency.	22 subjects	within-subjects design	Experimental Feedback	To reach a blue target on the panel. Positive or negative feedback is administered via a ‘GOOD’ or ‘BAD’ message. Three performance objectives: minimize the length of the reaching path, maintain touch contact for 3 seconds during reaching; and precisely hit the target's center.	● SOA: estimating time interval;<br>● Multidimensional Health Locus of Control (MHLC): scores for average internality (I), chance (C), and power of others (P);<br>● Contact accuracy (absolute error between the real location of contact and the center part of each target), reaching path length, and contact time.	● The agency gradually augments during the training. Training slope during PF is higher than NF ($p<0.01$);<br>● Decrease in path length following reward is significantly more pronounced ($p< 0.05$) than after NF;<br>● Although positive feedback (PF) leads to overall decreases in error for both the training slope and the post-training change compared to baseline, these decrements are not distinct from those observed with negative feedback (NF) ($p>0.05$);<br>● Both of the decreases in contact timing following PF is significantly greater than with NF ($p < 0.05$);<br>● No significant difference ($p > 0.05$) is observed comparing PF and NF for MHLC.

Nataraj et al., 2020b [32]	To prove a positive correlation between reaching performance and grasping agency, emphasizing the importance of incorporating agency judgment into movement rehabilitation practices.	16 subjects	within-subjects design	Mismatch	Participants control a virtual hand to execute reach-to-grasp movements mirroring their hand motions. They aim to optimize reach-to-grasp performance based on three criteria: making the reaching path length shorter, synchronizing hand reaching velocity with speed pacers, and completing the reach-to-grasp action within a precise 4-second timeframe. Additionally, participants must ensure consistent grasping of the target sphere with their thumb and index finger at specified locations. Also, they are required to estimate time intervals during the task.<br><br>Each participant engaged in the reach-to-grasp task using six distinct control modes:<br><br>• Baseline (virtual hand moves in proportion to the real hand's movements across all three dimensions);<br><br>• Grasp-Only (virtual hand is positioned near the target sphere requiring no reaching motion; only a grasp action is necessary to complete the trial);<br><br>• Slow (virtual hand moves 50% slower compared to the real hand's movements);<br><br>• Fast (virtual hand moves 50% faster than the real hand);<br><br>• Noise (virtual hand is infected by mild to moderate noise);<br><br>• Auto (virtual hand is progressively under automatic control).	• Intentional Binding: participants control a virtual hand to perform reach-to-grasp through movements of their hand;<br><br>• Questionnaire self-report (the degree to which the visualized hand motions reflect their intentions).	• Significant reduction in reaching performance between the Baseline test block from start to end of the session ($p=0.02$) is observed;<br><br>• The reach phase decreases the agency of grasp compared to the grasp-only test case ($p=0.017$);<br><br>• No differences ($p<0.05$) regarding explicit agency are detected across control modes;<br><br>• Inverse relationship between implicit and explicit agency outcomes across subject-mode pairs ($p<0.05$):<br><br>• Consistent differences in both agency ($p<0.001$) and performance ($p<0.0001$) control modes are observed;<br><br>• Trials characterized by high agency demonstrate significant reductions ($p<0.001$) in maximum path length, mean path length velocity, and maximum path length velocity;<br><br>• Positive association ($p<0.001$) is observed between agency and reaching performance across five distinct control modes

Ozen et al., 2019 [33]	To evaluate the potential of Model Predictive Controllers (MPC) for application in motor learning settings.	14 subjects	within-subjects design	Mismatch	Participants are supposed to swing the ball of a virtual pendulum to strike incoming targets using a robot. By grasping the end-effector (EE) of the robot, participants can visually and haptically interact with the pendulum.<br><br>The study comprises three conditions:<br>● "MPC - Free": Without any type of intervention;<br>● "eeMPC" (End-Effector MPC): MPC forces are applied at the end-effector of the robot, mimicking the participants' actions on the pendulum;<br>● "ballMPC" (Ball MPC): Forces are directly applied to the pendulum's red ball.	● Questions on the sense of embodiment and agency, selected from the Intrinsic Motivation Inventory (IMI);<br>● Kinetic metrics.	● No significant impact of the training modality on the average absolute error (p=0.11). However, the average errors for ballMPC, when normalized, are notably smaller compared to MPC-free;<br>● The training modality significantly affects the time integral of the absolute force in the given direction (p<0.001). This variable is higher during training with eeMPC compared to training with MPC-free and ballMPC;<br>● Training modality notably influences the perception of agency (p=0.016), with lower perceived agency during ballMPC than MPC-free;<br>● No significant effect of the training method is observed on any subscale of the IMI.
Padrao et al., 2015 [34]	- To dissociate neurophysiological mechanisms underlying the self-generated vs. externally imposed actions.<br><br>-To investigate an error detection mechanism that checks the coherence between final sensory feedback and expected sensory consequences of actions	Exp1= 18 subjects;<br><br>Exp2 = 9 subjects.	within-subjects design	Mismatch	EXP1<br><br>Respond in 1PP to an arrow pointing left or right while being surrounded by either compatible or conflicting flankers (Flanker Task).<br><br>The virtual hand movements are the same as the real ones(CM 40%), but in some trials they are different (InCM, 60%) in pseudo-random order, generating a "false (avatar) error."<br><br>EXP2	EXP1<br>● Correct responses; real errors (errors effectively committed by participants); and false errors (incongruence between real errors of the avatar and false error of participants);<br>● ERP signals related to self-generated errors and avatar errors are compared;	EXP1<br>● In InCM subjects are aware that errors caused by avatar are not attributed to their errors (p=0.0004);<br>● Q7 ("Sometimes the virtual hand seemed to be moving by itself") scores higher in the InCM condition than the CM (p=0.0003);<br>● Following false avatar errors, there is a correct reaction is

					Subjects observe the avatar's performance (Flanker Task) from a 1PP and have to count errors. Participants see a reproduction of their previous session.	• Questionnaire conditions on body ownership and agency.<br><br>EXP2<br><br>• Self-report questionnaires (experience, sense of body ownership, agency, and localization) and to determine whether the observed avatar's action is made by themselves;<br><br>• ERP analysis in InCM during the following conditions: correct responses toward the target; when avatar introduces false avatar errors; self-generated real errors;<br><br>• Hand tracker movement: correct, error, corrected (adjusted by subject), no response.	slower compared to correct trials ($p< 0.001$);<br><br>• Amplitude of Ne/ERN (frontal-central error-related negativity) is significantly enlarged for real errors than false avatar error trials ($p<0.0001$). The amplitude of N400 in avatar errors is negatively correlated with the subjective strength of virtual body ownership ($p<0.009$). Neurophysiological dissociation between I-eml (internal error-monitoring loop) and E-eml (external error-monitoring loop). Parietal distribution of N400.<br><br>EXP2<br><br>• Reduced subjective body ownership than in EXP1 CM condition ($p=0.042$). Participants show that the absence of visual-motor synchrony reduces ownership;<br><br>• Proprioceptive consistency of hand localization item is significantly diminished than in EXP1 CM condition ($p<0.007$);<br><br>• Analysis on (Q5 "my movements" vs. Q7 "not my movements") shows significant differences ($p=0.012$);<br><br>• Corroborated difference in neurophysiological signatures

							associated with avatar errors: I-eml (Ne/ERN; 60-150 ms) and E-eml (N400).
Ratcliffe and Newport, 2017 [35]	To assess the relative impacts of congruency in visual, temporal, and spatial information on body perception and the sense of self.	39 subjects	within-subjects design	Mismatch	Participants are supposed to reach and point to the green cross using the index finger in a manner such that the finger, if visible, aligns with the center of the cross. Participants view their unmanipulated right hand in the MIRAGE system. Then place their (unseen) hand inside MIRAGE. The experimenter moves the hand to a designated starting position, which changes depending on the condition. Participants tap their right index finger in synchronization with a beat. During synchronized tapping, the MIRAGE-mediated view displays two images of the right hand to the participant. In each trial, one hand moves synchronously with the subject's movements, while the other hand moves asynchronously. Additionally, one hand appears normal, while the other hand is distorted. Furthermore, one hand is presented in its real spatial location, while the other hand is presented in a false spatial location.<br><br>Conditions are completed in a pseudorandom order.	● Questionnaire on embodiment of hand under normal conditions (ownership, location, agency);<br><br>● Pointing task (mean distance reached, and accuracy).	● Ownership scores are higher when the veridical hand is synchronous (in the same spatial location as the participant's hand($p<0.001$). This pattern is reversed when the veridical hand is not synchronized ($p<0.001$);<br><br>● Agency scores are higher when the veridical hand is synchronous ($p<0.001$) The reverse pattern is shown when the veridical hand is not synchronized ($p<0.001$);<br><br>● When the veridical hand is normal, participants attribute greater agency scores to the real hand compared to when it is displaced ($p<0.001$);<br><br>● There are no notable differences in agency scores when the veridical hand is distorted ($p=0.615$);<br><br>● When the veridical hand is synchronized, judgments of location are higher than the veridical hand ($p<0.001$). Conversely, there are no significant differences in location scores when the veridical hand is asynchronous ($p=0.754$);<br><br>● The pointing task reveals significant main effects of both synchrony ($p<0.001$) and appearance.

Rognini et al., 2012 [36]	To measure the integration between visual and tactile integration through cross-modal congruency<br><br>effects (CCEs) in tasks involving bimanual movement using a robotic platform.	Exp1= 11 subjects;<br><br>Exp2= 12 subjects;<br><br>Exp3= 12 subjects.	within-subjects design	Mismatch	The robotic system is a bimanual haptic interface. On a visual display, movements, virtual objects, and virtual hands are shown in real-time. Visual distractors appear on either the same or opposite side as the tactile stimulus.<br><br>EXP1<br><br>Participants are instructed to keep their hands still to investigate potential observation of CEEs within the robotic setup.<br><br>EXP2<br><br>Participants repeat slow clockwise circular movements in the frontal plane. They are trained for both the static and the movement conditions.<br><br>EXP3<br><br>Participants repeat slow clockwise circular movements in the frontal plane. They are trained for synchronous conditions. At the end, they complete a questionnaire assessing agency for the observed movement and ownership of the seen hands.	• CCE analysis: only trials with correct responses and with RTs (reaction times) between 200 ms and 3000 ms are considered;<br><br>• Movement analysis: average velocity and trajectory norm (Euclidean norm of the three-dimensional trajectory between trials);<br><br>• Likert scale: items on agency for the seen movement and ownership of the seen hand.	EXP1<br><br>• CCEs (incongruent errors minus congruent errors) are notably greater when the visual distractors appear on the same side as the tactile stimulus compared to conditions where they appear on different sides ($p<0.03$);<br><br>• the CCE magnitude is greater in the same-side conditions than in the different-side conditions;<br><br>• RTs: a main effect of congruency ($p<0.00001$) and a significant interaction between side and congruency ($p<0.00001$).<br><br>EXP2<br><br>• RTs: significant main effect of congruency ($p < 0.001$) and a notable interaction between side and congruency ($p < 0.0001$), indicating that CCEs are notably more pronounced in the same-side conditions compared to the different-side conditions ($p < 0.0001$);<br><br>• Significant interaction between movement and congruency ($p<0.02$), driven by a notable difference between the static congruent condition and the movement congruent condition ($p < 0.0002$);<br><br>• CCEs shown in the static conditions are notably larger than those in the movement conditions ($p < 0.02$).

							EXP3<br><br>● RTs: a main effect of congruency (p<0.0001) and a significant interaction between side and congruency (p<0.001), confirming that CCEs are notably larger in the same-side conditions concerning the different-side conditions (p<0.0001);<br><br>● Significant three-way interaction among synchrony, side, and congruency (p<0.03), driven by a significant difference between the synchronous and asynchronous movement conditions only for the same-side congruent condition (p<0.001);<br><br>● The amplitude of CCEs in the same-side condition is greater in the synchronous than in the asynchronous case (p< 0.03);<br><br>● Ownership and agency are perceived significantly more strongly in the synchronous condition compared to the asynchronous condition (p<0.01).
Tidoni et al., 2014 [37]	To investigate the effectiveness of accurate auditory feedback (footsteps sounds) in the BCI-driven control of a humanoid robotic surrogate	28 subjects	within-subjects design	Mismatch	Participants are required to remotely control a humanoid robot through BCI.<br><br>There are four SGs:<br><br>● SG1: Steering the robot from the initial position to a table.	● Questionnaire on the quality of their interaction with the robot;<br><br>● EEG recordings;<br><br>● Walking time;	● Absence of learning effect on performance time (p=0.27);<br><br>● A significant main effect of Footstep (p=0.01), indicating faster performance time in the Synchronous condition compared to the Asynchronous condition.

					• SG2: Directing the robot to grasp a bottle.<br><br>• SG3: Maneuvering the robot as close as possible to a second table.<br><br>• SG4: Trying to drop the bottle as accurately as possible within a target area marked by two concentric circles.<br><br>Four conditions: "Synchronous" (hearing footsteps sound synchronous with the real footsteps of the robot), "Asynchronous"(hearing footsteps sound asynchronous with the robot), "Mirror Present" (seeing the robot's body reflected in a mirror), "Mirror Absent" (without seeing the robot's body).<br><br>There are four conditions:<br><br>• "Synchronous": Participants hear footstep sounds synchronized with the footstep of the robot;<br><br>• "Asynchronous": Participants hear footstep sounds not synchronized with the footstep of the robot.<br><br>• "Mirror Present": Participants see the reflex of the robot's body in the mirror;<br><br>• "Mirror Absent": Participants do not see the robot's body.	• Place accuracy.	However, there is no significant main effect of Mirror (p=0.09);<br><br>• No significant differences are detected in Place accuracy (p=0.16);<br><br>• No observed variations between experimental conditions regarding the sense of agency (p=0.38).
Weibel et al., 2015 [38]	To examine whether the feeling of agency might be associated with subtle	Exp1= 12 subjects;	within-subjects design	Mismatch	To perform some vertical pointing actions on a virtual surface using a haptic robot, which is sometimes perturbed via a small	• Analog visual scale for the feeling of control;	EXP1

	distortions in the haptic feedback.	Exp2= 12 subjects.			temporal delay (15 or 65 ms), and the next movement is started after an auditory cue.<br><br>• "Single change": the changes in pointing action parameters when the haptic delay is introduced(36 runs of 15 trials). Distortion (delayed haptic feedback) is introduced unpredictably.<br><br>• "Multiple changes": 5 trials, then participants report their feeling in control.<br><br>• Third session: to measure the detection threshold of the haptic feedback delay.<br><br>Participants are notified that haptic feedback may occasionally be delayed. They perform two trials each, where they believe they are comparing the levels (whether they are the same or different) of two consecutive surfaces in VR.<br><br>EXP1<br><br>Implicit and explicit distortions of the haptic feedback.<br><br>EXP2<br><br>Implicit distortions.	• Trajectories analysis: duration of deceleration phase and the amplitude of the deceleration (period during which participants slowdown in anticipation of the expected contact with the reference surface, that corresponds to the height at which participants initiate deceleration).	• No significant effect of the distortion on the amplitude of deceleration;<br><br>• Significant interaction between haptic delay and rank (degree of distortion) when compared with the condition without distortion ($p<0.001$);<br><br>• Higher perceived feeling of control when no distortion occurred, as compared to other conditions (1, 2, 3 transitions with $p<0.01$, <0.001 and <0.005, respectively).<br><br>EXP2<br><br>• A significant effect of rank on the amplitude of deceleration ($p<0.05$), and deceleration is lower in the trial following distortion;<br><br>• The perceived feeling of control with distortions is significantly lower than in conditions without distortion ($p<0.001$);<br><br>• The feeling of control reaches its peak when the duration of deceleration is prolonged ($p<0.001$) and is higher than when this duration is moderate or short ($p<0.005$).
Ozen et al., 2021 [39]	To study the efficacy of using Model Predictive Controllers (MPCs) as assistance strategies in facilitating the acquisition	40 subjects	between subjects design	Experimental Feedback	Participants in the study are required to manipulate a virtual pendulum to hit incoming targets using a haptic interface. They are assigned randomly to one of four training groups: Control, End-effector MPC	• Intrinsic Motivation Inventory (IMI) and questionnaire on embodiment;	• The use of assisting forces in the HG and eeMPC groups augments the movement variability compared to the

	of skills for complex dynamic tasks.				(which applies assisting forces on the end-effector), Ball MPC (which applies forces on the virtual pendulum ball to further reduce assisting forces), and Haptic Guidance (HG).<br><br>FIRST SESSION<br><br>• Baseline test block: participants have to hit the targets as close as possible;<br><br>• Training blocks: a robot assists participants allocated in the eeMPC, ballMPC, and HG groups, while those in the control group solely experience the haptic rendering of the pendulum dynamics., The assistance provided is removed unexpectedly in eight out of the 32 training blocks;<br><br>• Short-term retention test (STR) as the baseline.<br><br>SECOND SESSION<br><br>• Long-term retention test (LTR) and a second transfer test (with the same structure as in the first session). The order of targets in the training blocks varies between blocks but remains consistent across participants. Throughout the tests and training blocks, the average score is visually displayed to participants, to enhance their motivation.	• Hitting performance;<br><br>• Interaction forces (between the participants' hands and the robot end-effector);<br><br>• Assisting forces.	ballMPC and Control groups ($p<0.001$);<br><br>• The HG group exhibits higher interaction forces compared to other groups ($p<0.001$). Interaction forces are higher in the eeMPC group compared to the Control and ballMPC groups;<br><br>• Assisting forces provided by the haptic guidance controller are higher than those applied by other training methods ($p<0.001$). The eeMPC group also has significantly higher assisting forces compared to the Control and ballMPC groups;<br><br>• Significant interaction effect between the training group and the application of the assisting forces ($p<0.001$). The increase in the score when assistance is applied is notably higher in the ballMPC group compared to all other training groups.;<br><br>• The eeMPC group showed a significant improvement in task performance compared to the Control group and somewhat more than the HG group, though the difference is not statistically significant ($p = 0.09$)<br><br>• Positive association between the sense of agency and the learning amount, particularly notable in the eeMPC group ($p=0.07$);

							• No significant differences between training strategies across any subscale of the IMI.

1PP first person perspective; BOLD: blood-oxygen-level-dependent imaging; CCEs: cross-modal congruency effects; CM: congruent movement; EEG: electroencephalogram; E-eml: external error-monitoring loop; ERP: event-related potential; EVR: embodied virtual reality; fMRI: functional magnetic resonance imaging; HG: haptic guidance; I-eml: internal error-monitoring loop; IMUs: inertial measurement units; IMI: intrinsic motivation index; InCM: incongruent movement; JOA: judgment of agency; JOP: judgment of performance; LTR: long-term retention; MA: motor awareness; MAI: motor awareness index; MHLC: multidimensional health locus of control; MOVE_e: execute a previously observed finger sequence; MOVE_h: execute the observed sequence; MP: motor performance; MPC: model predictive controller; Ne/ERN: error-related negativity; NF: negative feedback; OTI observe to imitate; PF: positive feedback; RTs: reaction times; SGs: subgoals; SOA: sense of agency; STR: short-term retention; VE: virtual environment; VPE: velocity profile error; VR: virtual reality; WATCH_e: observe a non-anthropometric object
